# Correlates of stunting among under-five children in Bangladesh: a multilevel approach

**DOI:** 10.1186/s40795-019-0304-9

**Published:** 2019-10-07

**Authors:** Papia Sultana, Md. Mahfuzur Rahman, Jahanara Akter

**Affiliations:** 1 Department of Statistics, University of Rajshahi, Rajshahi, Bangladesh; 2 Global Public Health Research Foundation, Dhaka, Bangladesh

**Keywords:** Child malnutrition, Stunting, Prevalence, Multilevel generalized linear regression, Odds ratio, Bangladesh

## Abstract

**Background:**

Child malnutrition still remains a major cause of childhood morbidity and mortality in Bangladesh. This study aims to determine the prevalence and identify the associated risk factors of child malnutrition in Bangladesh using multilevel logistic regression model on data from the Bangladesh Demographic and Health Survey (BDHS), 2014.

**Methods:**

A total sample of 6965 children aged 0–59 months was extracted from BDHS 2014. We performed descriptive analysis and multilevel generalized linear regression analysis with clustered data structure.

**Results:**

Our findings show that among children the prevalence of moderate and severe values was respectively: 25 and 12% for stunting; 11 and 3.1% for wasting; 25 and 7.9% for underweight. The probability of stunting increased with age, with highest rate among children aged 36–47 months, which was significantly higher than children aged less than 6 months (OR = 6.71, 95% CI = 4.46, 10.10). Female children are found to be 11% less likely to be stunted than male children (OR = 0.89, 95% CI = 0.78, 1.02). Children with birth interval less than 24 months were significantly more likely to be stunted than children of first birth by 36% (OR = 1.36, 95% CI = 1.11, 1.67). Mothers with a normal BMI were 16% less likely to have children with stunting compared to mothers who are underweight (OR = 0.84, 95% CI = 0.76, 0.93). Other factors which were associated with a higher risk of stunting included parents with lower educational levels, children from the poorest wealth index, and mothers aged less than 20 years as first birth.

**Conclusion:**

Government and non-government organization should generate effective program to aware women of reproductive age about adverse effect of short birth interval, and to aware parents about standard height and weight according to age and gender of children. Overall, necessary steps may be taken to make people educated and to reduce household wealth inequality to improve nutritional status of children.

## Background

Malnutrition is defined by the World Health Organization as any deficiencies, excesses, or imbalances in a person’s intake of energy and/or nutrients [1]. Child nutrition status is an imperative indicator of poverty in a population; and poverty, malnutrition and disease are intertwined each other [2, 3]. Malnutrition has been referred as the single greatest threat to the world’s public health, especially for the developing countries, by the World Health Organization [1]. The nutritional status of under-five children is the most sensitive indicator of a society, country as well as world public health status. Malnutrition is an underlying cause of 45% of child death- accounting for almost one-half of the global total of children’s deaths [4]. Rates of child malnutrition in Bangladesh are among the highest in the world, with rates of stunting affecting more than 54% of preschool-age children, underweight in 56% and wasting in 17% [5]. In Bangladesh, nutrition-attributable mortality rate in children is about 53 per 1000 live birth according to Bangladesh Demographic and Health Survey 2011 [6]. Survivors are left vulnerable to illnesses, stunted growth and intellectual impairment. The prevalence of stunting in children has been steadily decreasing, from 51% in 2004 to 37% in 2014 [7, 8]. However, the average annual rate of reduction (AARR) of stunting in Bangladesh is 2.7 which is much less than the required 3.9 AARR [9] to reach the global World Health Assembly (WHA) target to have 40% reduction in child malnutrition by 2025 [10]. At current rate, Bangladesh is not likely to achieve the WHA target for reducing childhood malnutrition.

To further enable decline of malnutrition rate in children and reach zero hunger goals as set by international targets, comprehensive preventive policies targeting evidence-based high-risk groups is crucial. Numerous studies have focused on child malnutrition in Bangladesh and/or it’s associated risk factors [11–39]. However, in studies assessing risk factors, most utilized standard logistic regression as the statistical model; few have used multivariate approach such as one study using Bangladesh Demographic and Health Survey (BDHS) data from 2011 [40], and multilevel analysis using BDHS data from 2007 [11]. This study clearly demonstrated the significant variation of child malnutrition to hierarchical factors such as residence and division. Therefore, to determine child malnutrition in correct level, the variation due to hierarchical factors should be adjusted in the model through method of multilevel analysis [41–44]. To fill this gap in literature using the most recent datasets, our study aimed to identify the significant risk factors of stunting among under-five children in Bangladesh using multilevel generalized linear regression model using BDHS 2014 data.

## Methods

### Sample selection

**Fig. 1:**
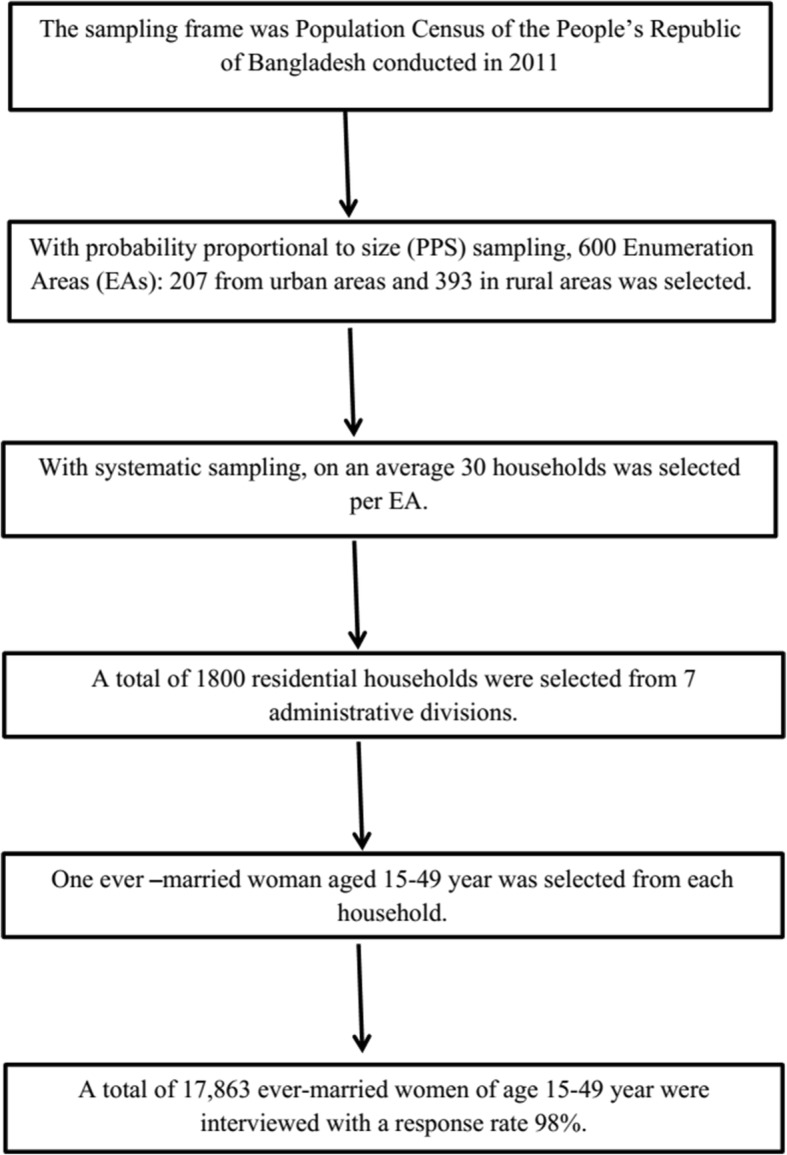
Study design of Bangladesh Demographic Health Survey, 2014

**Fig. 2:**
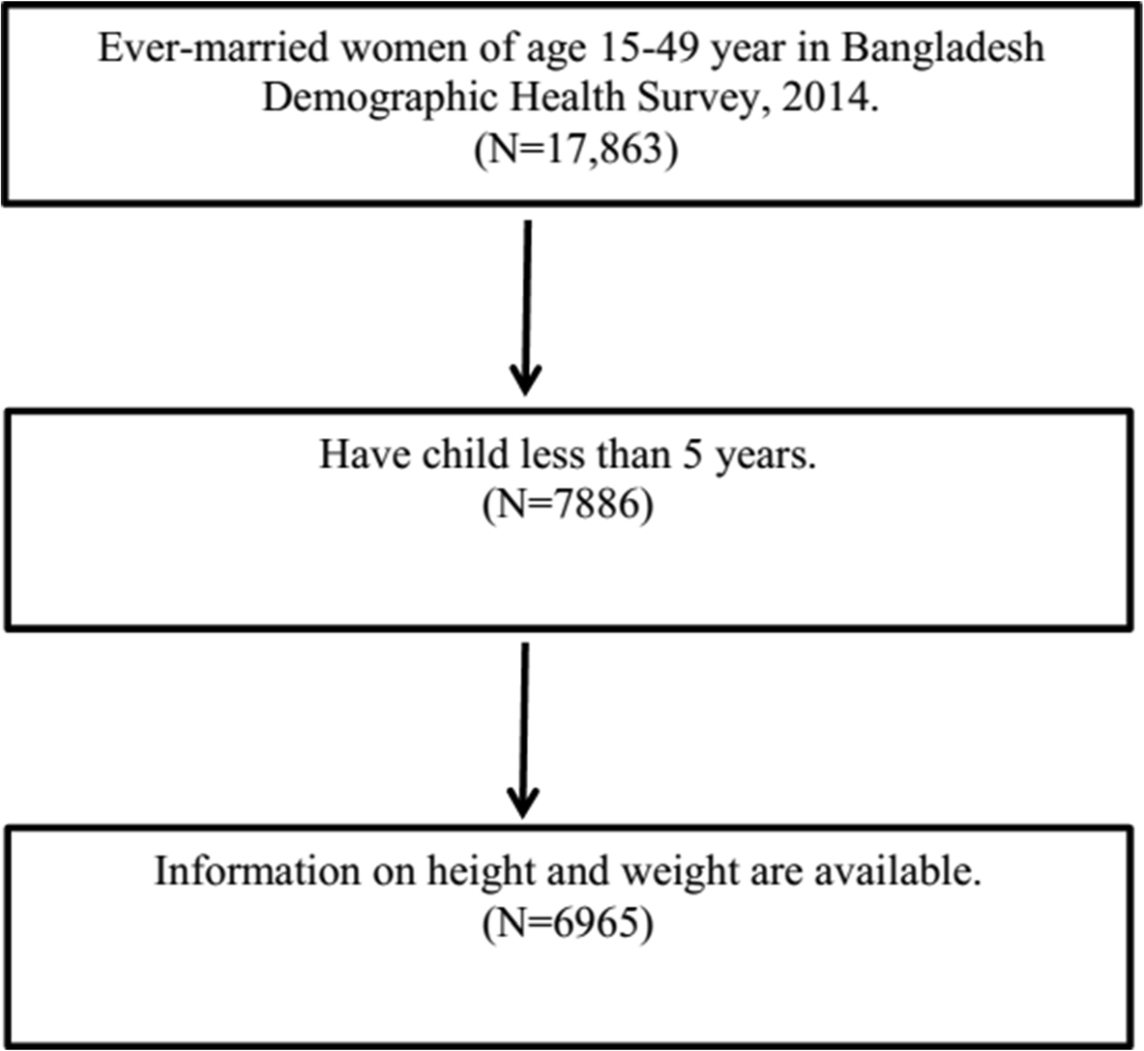
Data screening for under-five children in Bangladesh

The data used for the present study has been derived from BDHS, 2014, a nationally-representative survey conducted by the National Institute for Population Research and Training (NIPORT) of the Ministry of Health and Family Welfare [45]. The 2014 BDHS collected data on the nutritional status of children by measuring the height and weight of all children aged between 0 and 59 months in the selected households. The sample for the BDHS-2014 is nationally representative and covers the entire population residing in non-institutional dwelling units in the country. The survey used a sampling frame from the list of enumeration areas (EAs) of the 2011 Population and Housing Census of the People’s Republic of Bangladesh, provided by the Bangladesh Bureau of Statistics (BBS). The primary sampling unit (PSU) for the survey is an EA created to have an average of about 120 households. With the design, the survey selected 18,000 residential households, which were expected to result in completed interviews with about 18,000 ever-married women of age 15–49 years. A total of 17,863 ever-married women of age 15–49 years were interviewed, for a response rate of 98% (Fig. 1). Details about the survey collection method can be found in literature [46]. For the current study, the data has been screened for the under-five children for whom height and weight are available (Fig. 2). Therefore, the study uses a sample of size 6965 extracted from 7886 of BDHS 2014 data.

Three standard indices of physical growth that describe the nutritional status of children are: (i) Height-for-age, (ii) Weight-for-height, and (iii) Weight-for-age. Each of these indices provides different information about growth and body composition that can be used to assess nutritional status of under-five children. The nutritional status of children in the survey population is compared with the World Health Organization (WHO) Child Growth Standards to create z-scores [46] resulting the indicators as stunting, wasting and underweight. Standard deviations below the reference median of the WHO reference population in terms of height-for-age was defined as stunted (− 2 SD) or severely stunted (− 3 SD). Similarly, standard deviations below the reference median of the WHO reference population in terms of weight-for-age was defined as wasted (− 2 SD) or severely wasted (− 3 SD); standard deviations below the reference median of the WHO reference population in terms of weight-for-height was defined as underweight (− 2 SD) or severely underweight (− 3 SD). However, z-scores computed for the three health indicators height-for-age, weight-for-height, and weight-for-age were used in this study.

Size of child at birth or a child’s birth weight was recorded based on mother’s perception [46]. A child’s birth weight less than 2.5 kg was considered as less than average, and lager than 2.5 was considered as larger than average. Mother’s BMI was classified as underweight if it was less than 18.5 kg/m^2^, normal if it was between 18.5–24.9 kg/m^2^, overweight if it was between 25 and 29.9 kg/m^2^, and obese if it was above 30 kg/m^2^. For the educational level, primary level was defined as completing grade 5, secondary level was defined as completing grade 10. The wealth index was created in three steps. In the first step, a subset of household indicators (for example, access to safe drinking water, sanitation facilities, structure of housing, crowdedness of housing, type of fuel used for cooking, household possessions etc.) common to urban and rural areas was used to create wealth scores for households in both areas. Categorical variables were transformed into separate dichotomous (0–1) indicators. Those indicators and those that were continuous were then examined using a principal components analysis to produce a common factor score for each household. In the second step, separate factor scores were produced for households in urban and rural areas using area specific indicators. The third step combined the separate area-specific factor scores to produce a nationally applicable combined wealth index by adjusting area-specific scores through a regression on the common factor scores. The resulting combined wealth index had a mean of zero and a standard deviation of one. Once the index was computed, national-level wealth quintiles (from lowest to highest) were obtained by assigning the household score to each de jure household member, ranking each person in the population by his or her score, and then dividing the ranking into five equal categories, each comprising 20% of the population. Computations for wealth index were done by the authority of the survey and details can be found in survey literature [46]. In this study, stunting was used as the indicator of nutritional status of under-five children and used as the response variable in generalized linear regression analysis.

### Statistical analysis

We performed descriptive analysis to determine the characteristics of the study participants, which were presented with the frequency and percentage. Then, bivariate analyses were performed to compare child malnutrition (stunting, wasting and underweight) to the confounding variable. *P*-values had been obtained from bivariate simple linear regression to z-scores to check the association sex residence and division with child malnutrition. Lastly, multilevel generalized linear regression analysis had been performed with logit link and binomial family to identify significantly associated risk factors of child malnutrition. Various socio-demographic and economic variables were considered as risk factors. Division and residence of the respondents have been considered as the hierarchical variables. Two-level logistic regression (model-1 and model-2) and three level (model-3) logistic regression have been performed. In model-1 (two level) division has been considered as second level sources of variation; in model-2 (two level) residence has been considered as second level sources of variation; and in model-3 (three level) division has been considered as second level and residence has been as third level sources of variation. Statistical software StataSE version 13 (Stata Corp, USA) has been used to carry out statistical analyses. Missing values had been avoided in advanced analysis.

## Results

A total of 6965 children aged 0–59 months had been included in the study, of whom 3571 (51.3%) were male and 3394 (48.7%) were female (Table 1). A significant percentage (19.1%) of children was below average size at birth. Among the mothers 73% gave their first birth at before age 20 years and 22.4% mothers were underweighted. About 20.9% mothers had no antenatal visits during pregnancy and 15.4% mothers had no formal education. Among the respondents 68.6% were from rural area and 21.7% were of poorest wealth index.

**Table 1:** Characteristics of the study subjects, BDHS-2014

Characteristic	Total (sample size = 6965) Frequency(Percentage)	Male (sample size =3571)	Female (sample size = 3394)
		Frequency(Percentage)	Frequency(Percentage)
Age in months			
mean	29.56	29.42	29.72
< 6	558 (8.01)	302(8.46)	256 (7.54)
6–11	786 (11.28)	396 (11.09)	390 (11.49)
12–23	1456 (20.90)	744 (20.83)	712 (20.98)
24–35	1405 (20.17)	719 (20.13)	686 (20.21)
36–47	1377 (19.77)	701 (19.63)	676 (19.92)
48–59	1383 (19.86)	709 (19.85)	674 (19.86)
Size of child at birth			
Very large	96 (2.28)	55 (2.55)	41 (2.01)
Larger than average	456 (10.84)	251 (11.61)	205 (10.03)
Average	2849 (67.75)	1466 (67.84)	1383 (67.66)
Smaller than average	544 (12.94)	271 (12.54)	273 (13.36)
Very small	260 (6.18)	118 (5.46)	142 (6.95)
Live birth between births			
Yes	4 (0.09)	2 (0.09)	2 (0.10)
No	4261 (99.91)	2204 (99.91)	2057 (99.90)
Preceding birth interval (months)			
First birth	2700 (38.84)	1365 (38.31)	1335 (39.40)
< 24	476 (6.85)	243 (6.82)	233 (6.88)
24–47	1388 (19.97)	733 (20.57)	655 (19.33)
48 +	2387 (34.34)	1222 (34.30)	1165 (34.39)
Age of mother at 1st birth (years)			
< 20	5085 (73.01)	2608 (73.03)	2477 (72.98)
20–30	1838 (26.39)	940 (26.32)	898 (26.46)
30 +	42 (0.60)	23 (0.64)	19 (0.56)
Mother’s BMI			
Underweight	1556 (22.42)	797 (22.41)	759 (22.44)
Normal	4067 (58.60)	2096 (58.93)	1971 (58.26)
Overweight	1097 (15.81)	543 (15.27)	554 (16.38)
Obese	220 (3.17)	121 (3.40)	99 (2.93)
Frequency of antenatal visits during pregnancy			
No antenatal care visits	848 (20.95)	434 (20.80)	414 (21.11)
Antenatal visits	3196 (78.95)	1651 (79.11)	1545 (78.79)
Don’t know	4 (0.10)	2 (0.10)	2 (0.10)
Duration of breastfeeding			
Ever Breastfed	535 (13.15)	275 (13.13)	260 (13.18)
Never breastfed	46 (1.13)	22 (1.05)	24 (1.22)
Still breastfeeding	3481 (85.59)	1798 (85.82)	1683 (85.34)
Don’t know	5 (0.12)	0 (0.00)	5 (0.25)
Mother’s educational level			
No education	1076 (15.45)	588 (16.47)	488 (14.38)
Primary	1934 (27.77)	957 (26.80)	977 (28.79)
Secondary	3219 (46.22)	1631 (45.67)	1588 (46.79)
Higher	736 (10.57)	395 (11.06)	341 (10.05)
Mother’s employment status			
Yes	1747 (25.09)	885 (24.79)	862 (25.40)
No	5217 (74.91)	2685 (75.21)	2532 (74.60)
Father’s educational level			
No education	1736 (24.93)	875 (24.50)	861 (25.38)
Primary	2100 (30.16)	1047 (29.32)	1053 (31.04)
Secondary	2118 (30.42)	1119 (31.34)	999 (29.45)
Higher	1009 (14.49)	530 (14.84)	479 (14.12)
Father’s occupation			
Professional	651 (9.38)	335 (9.40)	316 (9.35)
Sales	1384 (19.94)	705 (19.79)	679 (20.09)
Agricultural-self employ	642 (9.25)	346 (9.71)	296 (8.76)
Agricultural-employ	1046 (15.07)	527 (14.80)	519 (15.36)
Services	583 (8.40)	272 (7.64)	311 (9.20)
Skilled manual	2460 (35.44)	1280 (35.93)	1180 (34.91)
Household and domestic	132 (1.90)	71 (1.99)	61 (1.80)
Don’t work	44 (0.63)	26 (0.73)	18 (0.53)
Division			
Barisal	812 (11.66)	416 (11.65)	396 (11.67)
Chittagong	1320 (18.95)	667 (18.68)	653 (19.27)
Dhaka	1213 (17.42)	620 (17.36)	593 (17.47)
Khulna	774 (11.11)	399 (11.17)	375 (11.05)
Rajshahi	875 (12.56)	442 (12.38)	433 (12.76)
Rangpur	865 (12.42)	456 (12.77)	409 (12.05)
Sylhet	1106 (15.88)	571 (15.99)	535 (15.76)
Type of Place of residence			
Urban	2188 (31.41)	1129 (31.62)	1059 (31.20)
Rural	4777 (68.59)	2442 (68.38)	2335 (68.80)
Wealth index			
Poorest	1515 (21.75)	794 (22.23)	721 (21.24)
Poorer	1307 (18.77)	674 (18.87)	633 (18.65)
Middle	1379 (19.80)	677 (18.96)	702 (20.68)
Richer	1420 (20.39)	732 (20.50)	688 (20.27)
Richest	1344 (19.30)	694 (19.43)	650 (19.15)

The data showed that among the children 12.2% were severely stunted, 24.9% were moderately stunted and 62.9% were well nourished (Table 2). It was found that 3.2% children were severely wasted and 11.7% were moderately wasted. About 8.3% of the children were found to be severely underweight, while 24.7% were moderately underweight.

**Table 2:** Prevalence of stunting, wasting and underweight of under-five children in Bangladesh, BDHS-2014

Category	Stunting (Height for age) Frequency (%)	Wasting (Weight for height) Frequency (%)	Underweight (Weight for age) Frequency (%)
Severely malnourished	833 (11.96)	213 (3.06)	552 (7.93)
Moderately malnourished	1714 (24.61)	792 (11.37)	1708 (24.52)
Normal (well-nourished)	4418 (63.43)	5960 (85.57)	4705 (67.55)

From Table 3, it had been found that stunting and underweight differ significantly to residence and division. It had been also found that rural children are more stunted than urban children and children of Sylhet division are mostly stunted. However, wasting differs significantly to gender, residence and division.

**Table 3:** Comparing nutritional status of under-five children to gender, residence and division, BDHS-2014

Category	Height for age (Stunted)			Weight for height (Wasted)			Weight for age (Underweight)		
	Severely malnourished Frequency (%)	Moderately malnourished Frequency (%)	Normal (Well- nourished) Frequency (%)	Severely malnourished Frequency (%)	Moderately malnourished Frequency (%)	Normal (Well- nourished) Frequency (%)	Severely malnourished Frequency (%)	Moderately malnourished Frequency (%)	Normal (Well- nourished) Frequency (%)
Sex									
Male	441 (12.35)	900 (25.20)	2230 (62.45)	132 (3.70)	411 (11.51)	3028 (84.79)	271 (7.59)	874 (24.47)	2426 (67.94)
Female	392 (11.55)	814 (23.98)	2188 (64.47)	81 (2.39)	381 (11.23)	2932 (86.39)	281 (8.28)	834 (24.57)	2279 (67.15)
*P*-value		0.092			0.008			0.329	
Residence									
Urban	224 (10.24)	479 (21.89)	1485 (67.87)	62 (2.83)	196 (8.96)	1930 (88.21)	147 (6.72)	445 (20.34)	1596 (72.94)
Rural	609 (12.75)	1235 (25.85)	2933 (61.40)	151 (3.16)	596 (12.48)	4030 (84.36)	405 (8.48)	1263 (26.44)	3109 (65.08)
*P*-value		< 0.001			< 0.001			< 0.001	
Division									
Barisal	83 (10.22)	225 (27.71)	504 (62.07)	34 (4.19)	107 (13.18)	671 (82.64)	60 (7.39)	212 (26.11)	540 (66.50)
Chittagong	186 (14.09)	315 (23.86)	819 (62.05)	41 (3.11)	152 (11.52)	1127 (85.38)	108 (8.18)	339 (25.68)	873 (66.14)
Dhaka	122 (10.06)	282 (23.25)	809 (66.69)	28 (2.31)	121 (9.98)	1064 (87.72)	81 (6.68)	253 (20.86)	879 (72.46)
Khulna	53 (6.85)	166 (21.45)	555 (71.71)	24 (3.10)	80 (10.34)	670 (86.56)	38 (4.91)	168 (21.71)	568 (73.39)
Rajshahi	82 (9.37)	182 (20.80)	611 (69.83)	32 (3.66)	118 (13.49)	725 (82.86)	59 (6.74)	218 (24.91)	598 (68.34)
Rangpur	87 (10.06)	216 (24.97)	562 (64.97)	32 (3.70)	105 (12.14)	728 (84.16)	74 (8.55)	209 (24.16)	582 (67.28)
Sylhet	220 (19.89)	328 (29.66)	558 (50.45)	22 (1.99)	109 (9.86)	975 (88.16)	132 (11.93)	309 (27.94)	665 (60.13)
*P*-value		< 0.001			0.095			< 0.001	

Table 4 represents odds ratio (OR) with 95% confidence interval (CI) obtained from multilevel generalized linear regression with binomial family and logit link to nutritional status of under-five children. From the results of Akaike Information Criterion (AIC), Bayesian Information Criterion (BIC), Intraclass Correlation Coefficient (ICC) and Median Odds Ratio (MOR), Model-III was the best. Our results showed that the odds of being stunting significantly increased with age, with highest OR in 36–47 months age group (OR = 6.71, 95% CI = 4.56,10.10). Children of preceding birth interval of less than 24 months was also significantly associated with increased odds of stunting (OR = 1.36, 95% CI = 1.11, 1.67). Conversely, female children was found to be 11% less likely to be stunted than male children (OR = 0.89, 95% CI = 0.78, 1.02). Other risk factors which significantly reduced the OR of stunting included children of mothers with normal BMI (OR = 0.84, 95% CI = 0.76, 0.93), higher educational level in mother or father (OR = 0.71, 95% CI = 0.53, 0.97 and OR = 0.61, 95% CI = 0.53, 0.70 respectively), and children of richest wealth index (OR = 0.42, 95% CI = 0.31, 0.57).

**Table 4:** Odds Ratio from multilevel generalized linear regression to stunting status of children in Bangladesh, BDHS-2014

Variable	Odds Ratio (95% Confidence Interval)		
	Model 1^a^	Model 2^b^	Model 3 ^c^
Age in months			
< 6 (RC)	1.00	1.00	1.00
6–11	1.94 (1.27–2.97)	1.89 (1.52–2.35)	1.94 (1.27–2.96)
12–23	5.08 (3.75–6.90)	4.91 (3.82–6.32)	5.09 (3.76–6.88)
24–35	5.87 (4.15–8.28)	5.72 (5.44–6.02)	5.88 (4.16–8.31)
36–47	6.71 (4.47–10.07)	6.55 (5.96–7.20)	6.71 (4.46–10.10)
48–59	4.47 (3.07–6.51)	4.40 (4.33–4.47)	4.48 (3.08–6.53)
Sex of child			
Male (RC)	1.00	1.00	1.00
Female	0.89 (0.78–1.02)	0.89 (0.85–0.93)	0.89 (0.78–1.02)
Preceding birth interval (months)			
First birth (RC)	1.00	1.00	1.00
< 24	1.36 (1.11–1.67)	1.47 (1.22–1.77)	1.36 (1.11–1.67)
24–47	1.15 (0.96–1.39)	1.25 (1.14–1.38)	1.16 (0.96–1.40)
48+	0.88 (0.77–1.02)	0.88 (0.83–0.93)	0.89 (0.77–1.02)
Age of mothers at 1st birth (years)			
< 20 (RC)	1.00	1.00	1.00
20–30	0.87 (0.77–0.98)	0.90 (0.77–1.06)	0.87 (0.76–0.98)
31 +	0.88 (0.48–1.59)	0.90 (0.53–1.53)	0.87 (0.49–1.56)
Mother’s BMI			
Underweight (RC)	1.00	1.00	1.00
Normal	0.84 (0.77–0.93)	0.83 (0.78–0.89)	0.84 (0.76–0.93)
Overweight	0.62 (0.51–0.76)	0.60 (0.54–0.67)	0.62 (0.51–0.76)
Obese	0.65 (0.46–0.93)	0.60 (0.49–0.73)	0.65 (0.46–0.92)
Mother’s educational level			
No education (RC)	1.00	1.00	1.00
Primary	0.96 (0.73–1.26)	0.93 (0.90–0.97)	0.96 (0.73–1.26)
Secondary	0.76 (0.60–0.96)	0.71 (0.61–0.83)	0.76 (0.61–0.96)
Higher	0.71 (0.52–0.97)	0.64 (0.39–1.03)	0.71 (0.53–0.97)
Mother’s employment status			
No (RC)	1.00	1.00	1.00
Yes	1.1 (0.99–1.24)	1.03 (0.89–1.18)	1.10 (0.98–1.23)
Father’s education level			
No education (RC)	1.00	1.00	1.00
Primary	0.85 (0.74–0.97)	0.85 (0.85–0.85)	0.85 (0.75–0.97)
Secondary	0.69 (0.58–0.82)	0.68 (0.57–0.79)	0.70 (0.59–0.82)
Higher	0.61 (0.52–0.70)	0.59 (0.51–0.68)	0.61 (0.53–0.70)
Father’s occupation			
Professional (RC)	1.00	1.00	1.00
Sales	1.04 (0.86–1.25)	1.02 (0.86–1.22)	1.03 (0.86–1.24)
Agricultural – self employ	1.06 (0.79–1.43)	1.03 (0.99–1.07)	1.06 (0.79–1.42)
Agricultural - employ	1.04 (0.79–1.37)	1.02 (0.94–1.11)	1.04 (0.79–1.36)
Services	1.22 (0.98–1.53)	1.24 (0.93–1.66)	1.22 (0.97–1.52)
Skilled manual	1.1 (0.82–1.46)	1.08 (0.84–1.39)	1.09 (0.82–1.45)
Household and domestic	0.72 (0.51–1.02)	0.81 (0.74–0.89)	0.72 (0.50–1.03)
Don’t work	1.16 (0.55–2.43)	1.23 (1.09–1.38)	1.16 (0.55–2.43)
Wealth index			
Poorest (RC)	1.00	1.00	1.00
Poorer	0.79 (0.63–0.99)	0.80 (0.78–0.82)	0.79 (0.63–0.99)
Middle	0.77 (0.70–0.84)	0.78 (0.74–0.82)	0.77 (0.69–0.85)
Richer	0.63 (0.49–0.82)	0.64 (0.41–1.00)	0.62 (0.47–0.82)
Richest	0.43 (0.32–0.57)	0.45 (0.31–0.65)	0.42 (0.31–0.57)
No. of observation	6900	6900	6900
AIC	8191.249	8243.575	8190.982
BIC	8464.82	8517.146	8471.393
Intraclass Correlation Coefficient (ICC)			
Division	0.0166945		0.0146549
Residence		0.0004238	0.0187098
Median Odds Ratio (MOR)			
Division	1.252		1.234
Residence		1.036	1.117
Random-effects Parameters Estimate (95% CI)			
Residence		0.04 (0.00–0.57)	0.12 (0.04–0.33)
Division	0.24 (0.13–0.42)		0.22 (0.11–0.44)
*P*-value	< 0.001	1.00	< 0.001

RC stands for ‘reference category’

^a^Model 1: two level generalized linear regression considering division as second level hierarchical factor

^b^Model 2: two level generalized linear regression considering residential status as second level hierarchical factor

^c^Model 3: three level generalized linear regression considering residential status as third level and division as second level hierarchical factors

## Discussion

In our multilevel analysis, we have found that children of age group 12–47 months, male, born with preceding birth interval less than 24 months, having underweight mothers, having mothers and fathers with lower education level and of poorest wealth index are more likely to be stunted.

Results from multilevel logistic regression show that as children grow up they become more likely to be stunted. This result is consistent with other studies in Bangladesh [17, 36] and also in neighboring countries: India [47], Nepal [48–50], China [51]. An increasing pattern of stunting by age is found to be undeviating with increasing pattern of communicable childhood diseases by age [52]. As discussed by Hong, Banta and Betancourt [23], this may partly be due to starting other foods along with breastfeeding to a child after 6 months of age, which increases the likelihood of taking polluted foods and minimizes the essential safety provided by breast milk. Moreover, children initiate crawling nearby at this age and are more probable to be carried out-of-doors, which makes them exposure to additional toxicities. Among the under five children, it is most likely to be stunted between age 12–47 months. Therefore, caring at early childhood is more likely to be protective and stunting becomes more prospective as the child becomes more dependent for caloric intake from foods which should be improved. More elaborative researches are needed to demonstrate in this regard.

Male children are found to be insignificantly more stunted than female. Gender has been identified a risk factor in many studies [25, 30, 33, 34]. Standard binary logistic regression (single level) also gave insignificant evidence about gender to be determinant of child malnutrition with BDHS-2014 data [17].

Coherent with other studies [26, 30, 33, 34] children of second birth order with less than 24 months birth interval are more likely to be stunted than children of first birth order and children of second birth order with more than 48 months birth interval. The association between stunting and second-order births with shorter interval may be due to competition for food within a household and mother may fail to take care two babies together within limited household.

Similar to our findings some studies confirmed association of child malnutrition with mother’s malnutrition [53]. In a traditionally patriarchal society such as Bangladesh, the availability and accessibility of nutritious foods for under-five children is highly dependent on the conduct of maternal duties and responsibilities towards their children which may be impaired when mothers are malnourished.

Education of parents is found to be a preventive factor of child malnutrition which is expected. Education always plays a positive role in health and diseases [37, 39]. Educated parents have more knowledge about health, nutrition, proper child care, health services, hygiene, proper food for children etc. Educated parents also contribute positively to promote the household income which is supportive to provide nutritional diet to their children. Moreover, Islam and his colleagues [54] discussed in this aspect that educated mothers are able to use insufficient family income and offered healthcare services efficiently, can minimize the family size, can sustain improved health stimulating behaviors and can afford better healthcare to their children [55, 56]. All these features may subsidize to keep children in virtuous nourishment.

Nutritional status is called a reflecting indicator of a family’s economic condition. This study also shows that children of families with poorest wealth index are most likely to be stunted, as found in other studies conducted in Bangladesh and in other countries [46–50, 57, 58]. This can be attributed to the fact that higher socioeconomic households have more ability to allocate necessary resources regarding nutrition for their children than poorer families. Reasonably allocation of more resources to their children improves their health conditions by reducing multiple health risks.

However, parent’s employment status does not found to have any significant role in managing child malnutrition.

Main strength of the study is that the data used in this study is a nationally representative, large sample size and enough information on child malnutrition. The study also has some limitations. The data don’t have information on some important factors like amount of diet given to children, mobility pattern of children etc. Some existed variables in the data including size of child at birth, antenatal visits, duration of breast feeding etc. are not included in the model due to a lot of missing information.

## Conclusion

In conclusion, this study clearly reveals that multilevel modeling should be used for hierarchical data to predict significant determinant child malnutrition in correct level. This study also has been identified using multilevel generalized linear regression model that under-five children who are male, of second birth order with less than 24 months, of underweight mothers, of lower educated parents and of poorest family are on high risk to be stunted. Therefore, policy makers and government should pay their attention more to take proper initiative to promote nutritional status in Bangladesh. Government may take necessary steps to aware women of reproductive age about adverse effect of short birth interval. Government and non-government organizations related to the health and nutrition should generate effective program to increase awareness of parents about the standard height and weight according to age and gender of the children. In addition, government may implement child care leave for employed parents with under-five children. Government also may establish childcare center with proper infrastructure, so that employed parents are not obligatory to leave their children at home under illiterate maid. Government may also encourage employed couples to stay with their older parents (or relatives) through some intervention to avoid the same. Overall, necessary steps may be taken to make people educated and to reduce household wealth inequality to improve nutritional status of children.

## Data Availability

Available at: https://dhsprogram.com.

## Abbreviations

AARR: Average Annual Rate of Reduction
AIC: Akaike Information Criterion
BDHS: Bangladesh Demographic and Health Survey
BIC: Bayesian Information Criterian
BMI: Body Mass Index
CI: Confidence Interval
ICC: Intraclass Correlation Coefficient
MOR: Median Odds Ratio
NIPRT: National Institute of Population Research and Training
OR: Odds Ratio
SD: Standard Deviation
WHA: World Health Assembly
WHO: World Health Organization
